# The MOBIS dataset: a large GPS dataset of mobility behaviour in Switzerland

**DOI:** 10.1007/s11116-022-10299-4

**Published:** 2022-06-21

**Authors:** Joseph Molloy, Alberto Castro, Thomas Götschi, Beaumont Schoeman, Christopher Tchervenkov, Uros Tomic, Beat Hintermann, Kay W. Axhausen

**Affiliations:** 1 IVT, ETH Zurich, Zurich, Switzerland; 2 WWZ, University of Basel, Basel, Switzerland; 3 INE, Zurich University of Applied Sciences, Winterthur, Switzerland

**Keywords:** GPS tracking, Transport pricing, External costs, Response rates, Mobility behaviour, Randomised controlled trial

## Abstract

This article presents the MOBIS dataset and underlying survey methods used in its collection. The MOBIS study was a nation-wide randomised controlled trial (RCT) of transport pricing in Switzerland, utilising a combination of postal recruitment, online surveys, and GPS tracking. 21,571 persons completed the first online survey, and 3680 persons completed 8 weeks of GPS tracking. Many continued tracking for over a year after the study was completed. In the field experiment, participants participated through the use of a GPS tracking app, Catch-my-Day, which logged their daily travel on different transport modes and imputed the trip segments and modes. The experiment lasted 8 weeks, bookended by two online surveys. After the first 4-week control phase, participants were split into two different treatment groups and a continued control group. An analysis of the survey participation shows that the technology is capable of supporting such an experiment on both Android and iOS, the two main mobile platforms. Significant differences in the engagement and attrition were observed between iOS and Android participants over the 8-week period. Finally, the attrition rate did not vary between treatment groups. This paper also reports on the wealth of data that are being made available for further research, which includes over 3 million trip stages and activities, labelled with transport mode and purpose respectively.

**Supplementary Information:**

The online version contains supplementary material available at 10.1007/s11116-022-10299-4.

## Introduction

Transport pricing is widely regarded as a promising policy measure to combat congestion, internalize external costs of transport, and offset decreasing fuel tax revenues. The concept of transport pricing was first proposed in the 1920’s as an example of a corrective tax to internalise congestion externalities (Pigou 1920). Despite the theoretical capabilities to maximise infrastructure utilisation, transport pricing has only been sparsely implemented in practice as it is typically viewed as a ‘new tax’ and is thus associated with strong political resistance. Schemes in London (Santos and Shaffer 2004; Leape 2006), Stockholm (Eliasson et al. 2009) and Singapore (Chin 2005; Agarwal and Koo 2016; Tan 2020) are three well-known examples where limited transport pricing has been implemented in the form of congestion charges: Cars entering the central business district during certain hours have to pay a fee. These ‘congestion charges’ do not reflect all the external costs from all modes of transportation. Schemes have also been implemented in a number of cities including Oslo, Milan, Paris, Rome and Stuttgart.

Within this context, we describe here the newly released MOBIS (MOBIlity in Switzerland) dataset, collected during the study of the same name. MOBIS was a trilingual, national-scale transport pricing survey and randomised controlled trial in Switzerland, combining traditional survey methods and app-based GPS tracking.

The study aimed to understand the effects on travel behaviour of (a) informing subjects about congestion, health effects, and carbon emissions of their mobility, and (b) actually charging subjects the external costs associated with these 3 factors under a transport pricing experiment. To do this, we examined two different treatments—information and pricing & information (pricing). In the current political discourse, it is of interest to understand if information measures are found to have a similar impact as transport pricing. On the other hand, evidence for pricing would support calls to restructure current mobility taxes and subsidies.

While the primary goal of the study was to investigate the changes in mobility behaviour under a transport pricing scheme, the non-treated portion of the dataset provides a wealth of data for more general mobility research.

We also take this opportunity here to report our experiences and lessons learned undertaking the MOBIS study (Axhausen et al. 2021), which we hope will be informative for other researchers aiming to undertake similar GPS-based studies. We present both the survey method and an analysis of the effectiveness of the app-based tracking. In particular, contributions include a detailed analysis of the response rate over the duration of the study, and how it was impacted by the differences between iOS and Android devices.

The paper is structured as followed. "Literature review" Section covers the relevant literature on GPS tracking and transport pricing. "Methodology" Section details the survey method, including recruitment, online surveys and GPS tracking. "Results and discussion" Section presents the meta-analysis of the survey method, including response rates, tracking attrition and participant engagement. "Conclusion" Section concludes.

## Literature review

The use of GPS tracking for mobility research is now widespread. Multiple studies have identified how traditional travel diaries under-report the number of trips, due to, among other reasons, response burden and memory recall (Janzen et al. 2018; Wolf et al. 2003; Stopher et al. 2007). Passive tracking mostly mitigates these issues, although the collecting of trip metadata such as detailed trip purpose, fellow passengers and travel expenses mostly still requires more traditional survey methods. Furthermore, the performance of GPS tracking depends on the quality of the GPS traces, and the algorithms used to identify trips, stages and activities, as well as the mode and purpose of travel. Here there has been significant advances in recent years (Schuessler and Axhausen 2009; Marra et al. 2019). For two comprehensive reviews on the processing of GPS tracking data, the reader is referred to Shen and Stopher (2014) and Nikolic and Bierlaire (2017). Other studies note that the performance of the algorithms is highly dependent on the quality of the GPS data (Montini et al. 2015; Harding 2019; Burkhard et al. 2020).

One of the key factors influencing the quality of GPS data is the device used. This can be either a dedicated GPS logger, or a smartphone, where the data is collected through an app. The quality of the data can vary between devices, in particular between iOS and Android devices, depending for example on battery saving settings.

Few studies have explored the implications of this iOS/Android dichotomy and the implications for mobility studies using app-based tracking. Harding (2019) compared the performance of trip identification and mode detection by different apps and found that iOS-based apps tended to have a higher accuracy. However, not only is the quality of the recorded data important, but also the attrition rate throughout the study, as this ultimately determines the sample size. This is an open question that has not been widely explored. The market penetration rates of iOS and Android—and even different Android-based manufacturers—varies across regions and, possibly, segments of the population. For studies requiring a representative sample, for example official national travel surveys, an understanding of these factors is important.

### Transport pricing studies

There has been much study of the topic of transport pricing, including the development mathematical theoretical bases (Small et al. 2004; Verhoef et al. 1996) and simulation experiments (Meyer de Freitas et al. 2017; Chakirov 2016; Kaddoura 2015). Most of the research and practical implementations have focused specifically on road pricing, which is a limited form of transport pricing that focuses on drivers.

Although there is evidence for the success of congestion pricing (Santos and Shaffer 2004; Leape 2006; Eliasson et al. 2009), understanding the effects of broader transport pricing schemes remains a challenge. A key challenge is understanding the potential impacts of the proposed policy. Multiple studies have looked at route, mode and destination choice within the context of various pricing schemes using stated-preference experiments (Vrtic et al. 2010; Washbrook et al. 2006; Li and Hensher 2012). Work on the acceptance of pricing schemes includes (Vrtic et al. 2007; Jakobsson et al. 2000). More recently, the proliferation of affordable GPS tracking and mobile connectivity has opened up the possibilities of field experiments exploring transport users’ behavioural responses under a pricing scheme, which would have been financially and logically infeasible in the pre-smartphone era. In one of the first examples, Nielsen (2004) equipped 500 cars with a GPS-based device, and monitored participants for a control period before exposing them to a pricing scheme for the Copenhagen region. This study was in the pre-smartphone era and hence limited to a small sample size and no control group. A similar study using car-based GPS loggers was performed in Melbourne, in which 1400 toll road users experienced different types of congestion charges (Transurban 2016; Martin and Thornton 2017). A period of several months was used to monitor baseline behaviour before the pricing schemes were introduced for three quarters of the sample. In both these experiments, only car trips with the primary household vehicle were tracked. Public transport and active modes were not recorded. The Melbourne study did investigate possible modal shifts to rail commuting, by identifying car trips and subsequent parking at railway stations. The study reported that 30% of participants reported changing their road travel use under the pricing scheme. Until now there have been no studies that have attempted to use smartphone-based GPS tracking to look at road or transport pricing, limiting the opportunity to understand modal shifts.

## Methodology

**Fig. 1:**
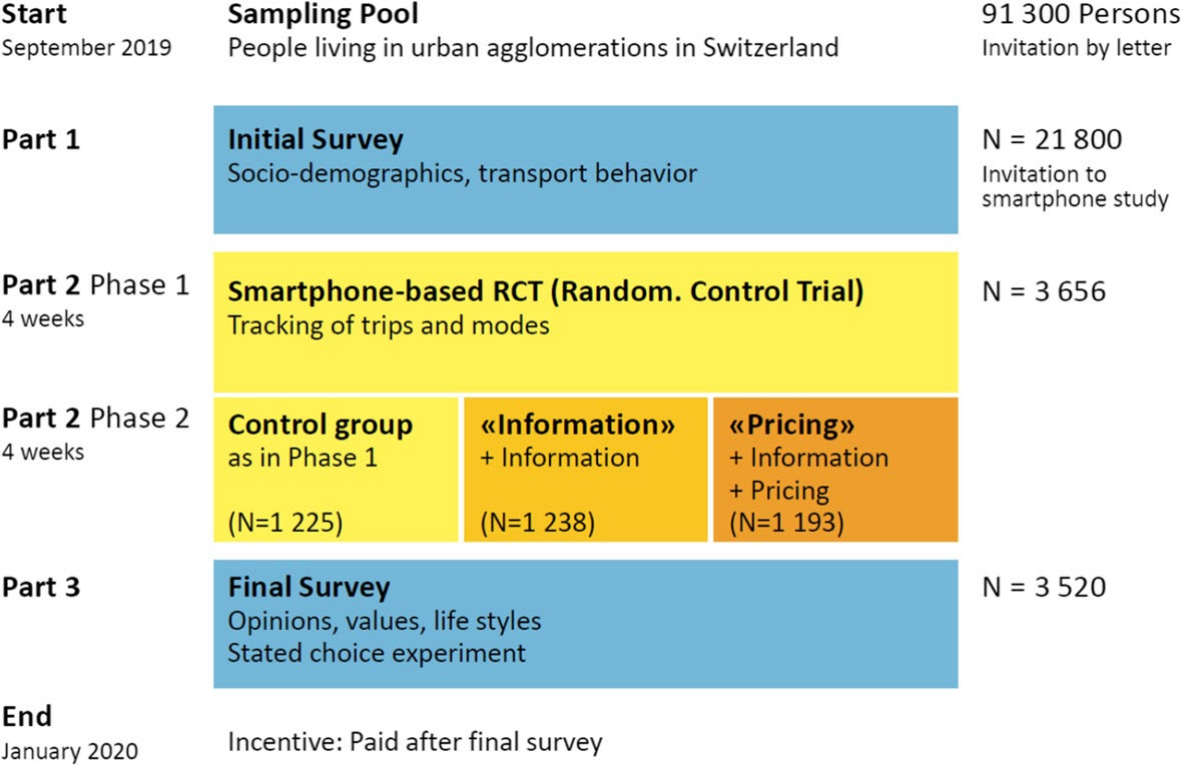
Overview of the MOBIS study design

The 8-week study consisted of two consecutive 4-week phases, a control and treatment phase respectively, bookended by introductory and concluding online surveys. The study recruitment started at the beginning of September 2019, and the last participants completed the study in January 2020. Figure 1 provides an overview of the study design.

### Experience gained from the pilot study

A pilot study with a mail-out sample of 1500 letters was undertaken to estimate the expected response rate for the main study and test the surveys and GPS tracking. This pretest had multiple goals:

- To determine the best recruitment method between sending up to three invitation letters and sending only one letter followed by a phone call
- To estimate the number of addresses required for the main study based on the participation rates observed in the pilot study
- To test the resilience of the planned recruitment and data collection system, including surveys, tracking app, and participant help desk, among others.

The pilot study took place between April and the end of July 2019. 1500 persons were invited to the pilot study by mail, using a sample of addresses and phone numbers purchased from a private vendor, Schober Information Group AG (renamed KünzlerBachmann Directmarketing SIG AG in 2020). Half the addresses (750) were used for the 3-letter method and the other half for the 1-letter method. Two weeks after the first invitation letter, if the invited persons did not respond (i.e. completing the introduction survey), a reminder was sent out. People assigned to the 3-letter group received up to two additional invitation letters and a phone call to kindly remind them to participate in the study. In contrast, addresses in the 1-letter group only received a phone call. The pilot study found that the 3-letter method was more effective for the recruitment.

Specifically, 28% of the 3-letter group completed the introduction survey, while only 15% of the 1-letter group did so. The recruitment rate of the letters slightly decreased over time. The first letter recruited on average around 11% of the recipients (9% in the 3-letter group and 12% in the 1-letter group), while the second letter recruited around 10% and the third one around 8%. The phone call contributed on average 2% of the called persons (around 3% in the 1-letter group and 1% in the 3-letter group). Based on these findings, the 3-letter approach without phone calls was chosen for the main study.

On average, 3.4% of the invited people (3.2% in the 1-letter group and 3.4% in the 3-letter group) completed the study, i.e., they filled out the introductory survey, qualified and registered for the field experiment, tracked during 8 weeks and filled out the final survey.

For the pilot study, the ETH-IVT Travel Diary app (Marra et al. 2019) was used to track the participants. While the app itself functioned well for collecting raw tracking data from the participants, the performance of the segmentation and mode detection was insufficient for real-world application, despite promising results during earlier testing. Due to the project’s tight time-frame, improving the machine learning algorithms before the latest possible start date of the main study was not feasible. Hence, the Catch-my-Day app (developed by MotionTag for a previous IVT study on carsharing) was chosen as an alternative for the main study. A further lesson learned from the pilot study was that an efficient help desk (per email and phone) was required for the main study. A help desk management tool (we used Freshdesk) and email templates were necessary to manage the high number of queries received from the participants.

### Initial recruitment

For the main study, a representative list of 60,000 addresses randomly selected across the major agglomerations (in the German and French speaking parts) of Switzerland from the Swiss Federal Statistical Office was used. Based on the response rate in the pretest, this address sample was skewed to account for under-represented groups. Additionally, to achieve the desired sample size of 3500 study participants, a second wave of around 30,000 persons were contacted using addresses from a private vendor, yielding a total of a little over 90,000 invitations. Only people living in an agglomeration area of Switzerland (excluding the Italian-speaking canton of Ticino) were invited to participate in the study.

The letter invited the recipients to fill in a screening-survey with transport-related questions and, if they met the inclusion criteria, to participate in a smartphone-based mobility experiment where they would receive 100 CHF (100 USD) for participating for the entire 8 weeks. Neither the “transport pricing” nature of the study nor the focus on the external costs of transport was shared with the participants.

Two reminder letters were also sent in the first wave, 4 and 7 weeks after the invitation letter was received, to those who had not responded to previous letters. No reminders were sent in the second wave as the target number of 3500 participants had already been achieved.

### Introductory survey

The initial survey was designed to determine a respondent’s eligibility for the main tracking study and collect data that would be needed in the calculation of external costs (such as mobility tool ownership, car type and age, and some general attitudes towards transport policies). The survey forms are provided in Online Appendix A.

The response burden for the initial survey was 183 points, based on the scheme presented in (Axhausen and Weis 2010; Schmid and Axhausen 2019). The aim was to keep the introductory survey extremely short, and only ask for the necessary information for assessing the eligibility of the participants and the required information on mobility tools required for the tracking.

### Recruitment for the RCT

The participants who completed the introductory survey were assessed against the eligibility criteria for the RCT. Specifically, participants

- Had to use a car at least two days a week (including as a passenger or with a taxi/Uber).
- Were restricted to the age of 18 to 65
- Must be able to walk without assistance
- Must own a smartphone
- Were not allowed to drive in a professional capacity—i.e. postman/woman or taxi driver.

Those who met the requirements for the study and gave consent to participate were sent an email with a unique registration code and a link to download the Catch-my-Day app and to participate in the tracking study.

### Randomised controlled trial

The 8-week study period was divided into two 4-week phases.

During the first phase, all study participants were treated equally, receiving weekly reports of their mobility behaviour by email, which included tracked distance by transport mode. During the treatment phase, the study participants received additional "treatments" beyond the weekly reports of the observation phase, depending on their randomly assigned group (pricing, information or control).

**Fig. 2:**
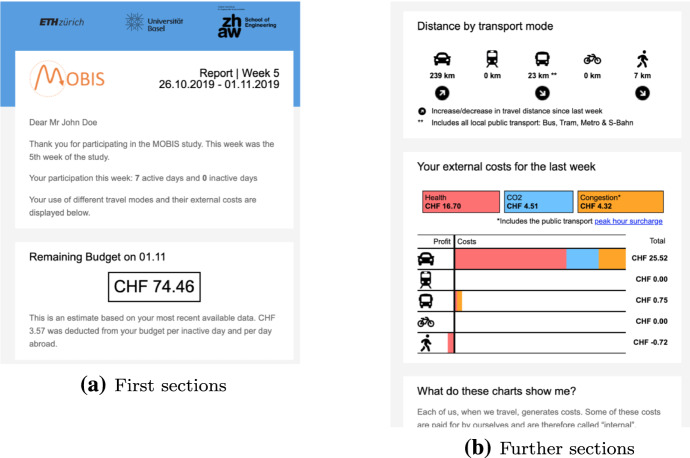
Weekly report to participants in the pricing group

At the beginning of the second phase, participants were randomly assigned to either the control group, or one of the two treatment groups. The information and pricing groups received additional information on the externalities they caused. Furthermore, participants in the pricing group were provided with a mobility budget, equal to 120% of their external costs in the first phase, from which their external costs in phase 2 were subtracted. The additional 20% were added to account for the possibility of participants increasing their external costs due to changes in their home or work location. The minimum budget was set to 50 CHF. Any balance remaining at the end of the 8 weeks was transferred to them as an incentive to reduce their externalities, in addition to the 100 CHF participation incentive. If the balance fell below zero, no additional money was transferred. Table 1 shows the descriptive statistics of the allocated budgets and remaining balances paid out to the participants. An example of the weekly reports is provided in Fig. 2.

**Table 1:** Descriptive statistics of allocated budgets and remaining balances in CHF

	Mean	std	Min	10%	25%	50%	75%	90%	Max
Allocated budget	171.61	101.17	50.00	60.00	95.00	150.00	225.00	310.00	745.00
Remaining balance	42.59	58.77	− 220.61	− 11.68	6.88	31.99	70.03	111.53	432.68
Amount paid out	47.01	52.58	0.00	0.00	6.88	31.99	70.03	111.53	432.68

The externalities were separated into health, environmental and congestion costs, which were computed using a data pipeline run every evening. For more details on the externality computation, please refer to Molloy et al. (2021b). The calculations are based on the HBEFA (Handbook Emission Factors for Road Transport), relevant Swiss norms and the IVT MATSim scenario for Switzerland (Hörl et al. 2019). Additionally, data collected from the introduction survey was incorporated into the data processing pipeline to improve the computation: Information on the participant’s main vehicle was used to calculate individualised external costs.

There may be unobserved determinants of transport behaviour (e.g. general traffic volume, road repairs, weather), which may have changed during the same time as we applied the pricing and information treatments. To assess such bias, the MOBIS study used a control group without any treatments which was observed simultaneously to the pricing and information groups. The control group obtained the same information about their travel as during the observation phase, i.e. a breakdown of distances travelled by mode, but without any information about associated costs.

### Tracking app

The Catch-my-Day app is a location tracker for iOS and Android, which uses the location services of the respective operating system. GPS tracks are stored on the phone and uploaded to the MotionTag analytics platform, where stages, travel modes and activities are imputed. The following modes are included in the Catch-my-Day app. Those marked with an asterisk are not automatically detected, but can be chosen by the user as a correction.

- Airplane
- Bicycle
- Boat*
- Bus
- Car
- Carsharing*
- Ferry
- Motorbike/Scooter*
- S-Bahn (Local train)
- Regional train
- Subway
- Taxi/Uber*
- Train (other)
- Tram
- Walk

Users can view their daily travel patterns on their phone in the form of a logbook, validate the travel mode and activity purpose or indicate if a trip or activity did not take place. The database stores both their correction and the original algorithmic imputation. There are some user-interface differences between the iOS and Android versions, which are most noticeable in the trip validation interface.

**Fig. 3:**
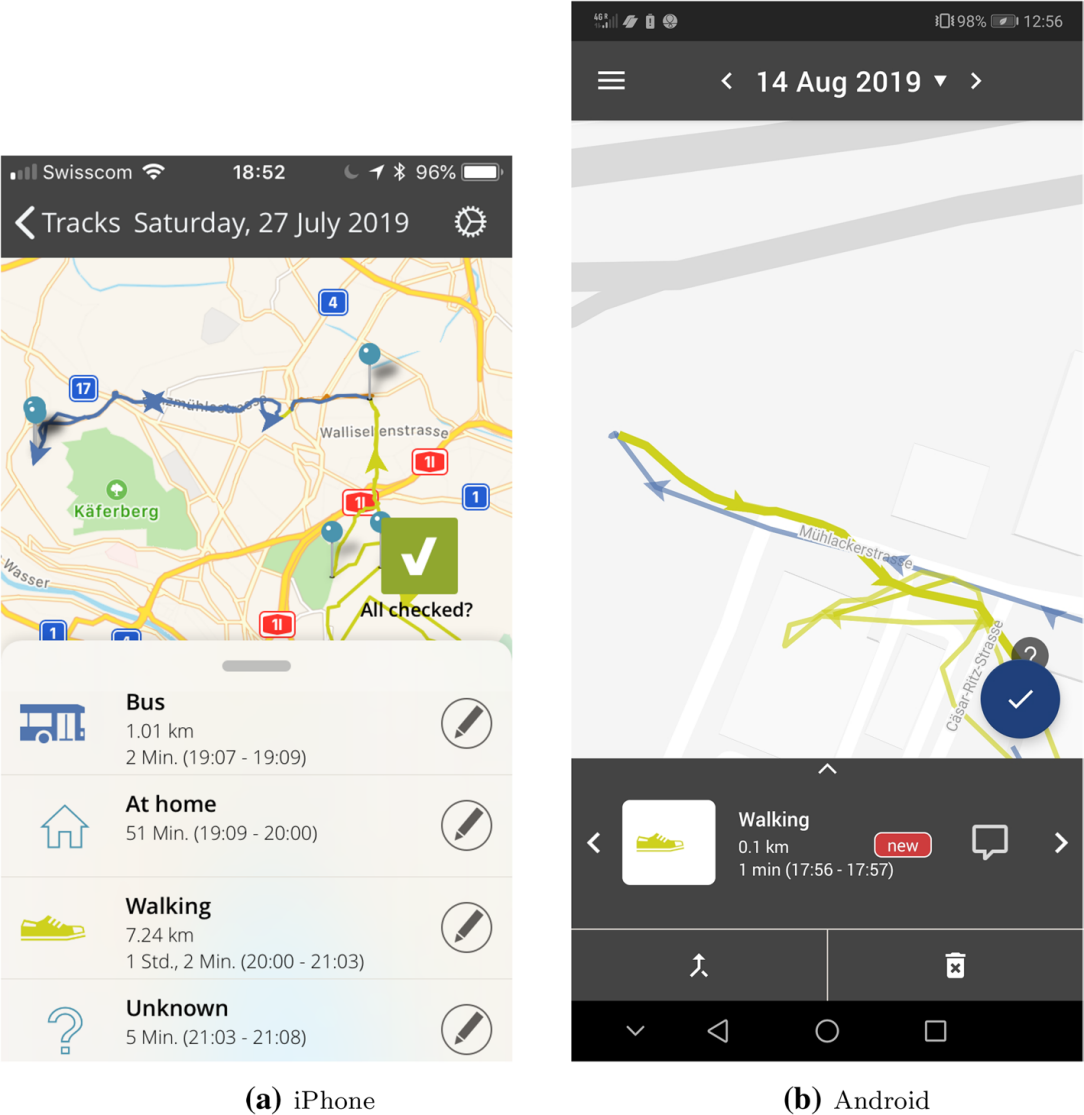
Trip/validation interface

Users could view their daily travel log in the app, and correct any incorrect travel mode imputations. Validation in the treatment phase was still allowed, even for the pricing group. Disabling validation in the treatment phase would have disadvantaged those affected by misdetection, especially if they had made corrections in the control phase, due to the lower external costs of public transport. To counter any possible ‘gaming’ of the experiment, an outlier analysis was performed before transferring the incentive to the participants. No clearly suspicious behaviour was observed, except for one participant who seemed to switch to riding his e-bike for the entire second phase of the study. Figure 3 presents the validation interface of the app for the respective operating systems.

Users were required to activate the app by creating an account, which required the provision of an email address and the choice of a password, along with the unique registration code provided. Participants were not required to validate their trips and activities, but were informed that this was possible and would be appreciated.

To increase the retention rate, automated reminder emails were sent to participants when they had not activated the app, or no data was recorded for a certain number of days. A help desk was set up for participants experiencing difficulties. User guides on how to correctly configure one’s smartphone for the app were provided. Additionally, participants who did not record data on at least 12 of the first 28 days were removed from the study, and notified by email.

### Final survey

The final survey included a series of stated-choice experiments and lifestyle and values questions, as well as awareness questions to gauge if participants understood the experiment and were therefore ‘knowledgeable’ participants. Completion of the final survey was a condition for receiving the incentive.

For the final survey, the response burden was allowed to be larger (471.5 points), as the participants were required to complete the final survey, which included a battery of stated-preference questions on mode-choice, before they could receive the incentive for their participation in the tracking. Hence there was less concern about the response rate.

### Post-experiment tracking

On completion of the 8 weeks of tracking, participants were informed that they could uninstall the app if they desired. However, they were also invited to continue tracking, albeit with no further financial incentive. The aim here was to hopefully have a sub-sample allowing the study of the persistence of the treatments after the study was completed.

At the start of the Covid-19 pandemic in March 2020, there were still around 300 participants still tracking post-experiment. With the obvious implications of the proposed lockdown measures on mobility behaviour, the whole panel of completed participants (3680) was invited to reactivate the tracking. Around one third chose to do so, and the tracking data from this ’restart’ is also included in the dataset, for the first year of the pandemic. Additional online surveys were conducted to update the participants information, especially concerning work status, working-from-home and mobility tools. These updates are also provided with the dataset, and a first analysis of mobility behaviour during the pandemic can be found in (Molloy et al. 2021a).

### Analysis methods

In the analysis of the tracking attrition, survival analysis is used. In particular, two modelling approaches are used, Kaplan-Meier and the Cox proportional-hazards model. Both methods are used to analyse the duration until an event occurs, traditionally death in medical research. Here, these methods are applied with the event *E* being the drop-out of the participant from the study, measured as the date of the last tracking point recorded.

The Kaplan-Meier estimator (Kaplan and Meier 1958) is a non-parametric method of estimating the survival function—in this paper as the percentage of participants who remain tracking after a certain period of time. The Cox proportional-hazards model (Cox 1972) on the other-hand is a regression model which investigates the association between the survival time of patients and one or more predictor variables.

## Results and discussion

In this paper, we present the results in terms of participation and the collection of tracking data. The analysis of the field experiment is still ongoing and will be presented elsewhere.

### Sample demographics

Table 2 shows the socio-demographic characteristics of the participants of the introduction survey and the tracking study and compares them to the Swiss Mobility and Transport Microcensus (MTMC), which is a representative survey of the Swiss population (BFS and ARE 2017).

The MOBIS tracking study imposed an eligibility criterion related to car use, among others. The respondents of the MOBIS introduction survey differ from the MTMC population in terms of the age distribution, as we limited the study to ages 18–65, and the regional coverage (only urban agglomerations and excluding the canton of Ticino). The MOBIS sample also has higher levels of education, employment and income.

The tracking sample differs from the introduction survey sample in terms of employment, household size income, and access to car, due to the eligibility requirement of travelling by car on at least two days per week. This condition is correlated with working away from home, which in turn drives the differences in the other variables. The cantons of Vaud (19% of the tracking sample) and Geneva (9%) account for the vast majority of the French-speaking participants, whereas the German-speaking participants mostly come from Zurich (38%), Basel (2% city and 10% region), Aargau (5%) and Bern (12%).

**Table 2:** Demographic information for the MOBIS sample

Variable	Level	MOBIS Intro Full	MOBIS tracking	MTMC
Age	[18, 25]	20.1	19.4	14.3
	(25, 35]	19.4	17.9	21.4
	(35, 45]	19.9	22.5	22.6
	(45, 55]	21.6	23.2	23.7
	(55, 65]	19.0	17.0	17.9
Education	Mandatory	9.2	6.6	13.8
	Secondary	43.3	48.5	47.5
	Higher	47.5	44.9	38.7
Employment	Employed	68.7	72.2	68.8
	Self-employed	7.3	6.3	8.8
	Apprentice	1.9	1.7	2.2
	Unemployed	4.4	4.0	3.9
	Student	9.3	8.0	3.0
	Retired	2.5	2.3	3.6
	Other	5.9	5.5	9.7
Gender	Male	48.9	49.7	49.4
	Female	51.1	50.3	50.6
Household size	1	15.5	11.7	18.3
	2	31.7	29.8	32.0
	3	20.5	21.5	19.9
	4	23.6	27.7	20.7
	5 or more	8.6	9.3	9.1
Income	4000 CHF or less	12.2	7.4	8.8
	4001–8000 CHF	29.4	29.4	31.4
	8001–12,000 CHF	24.5	29.2	24.6
	12,001–16,000 CHF	12.1	14.6	11.7
	More than 16,000 CHF	8.0	9.9	8.4
	Prefer not to say	13.8	9.5	5.8
	Don’t know			9.2
Language	German	62.7	66.0	69.5
	French	28.6	26.4	26.5
	Italian			4.0
	English	8.7	7.7	
Nationality	Switzerland	98.4	98.1	69.5
	Other	1.6	1.9	30.5
Access to car	Yes	61.0	87.7	69.7
	Sometimes	15.5	11.1	22.7
	No	23.5	1.2	7.5
Full PT subscription	Yes	37.2	23.9	34.5
	No	62.8	76.1	65.5
Half fare PT subscription	Yes	47.6	48.6	37.6
	No	52.4	51.4	62.4
No PT subscription	Yes	26.0	33.6	37.9
	No	74.0	66.4	62.1
Access to bicycle	Yes	68.5	71.3	70.1
	Sometimes	4.1	4.6	8.8
	No	27.4	24.1	21.1

Descriptive statistics shown for the MOBIS introduction survey (N = 20,783), the MOBIS tracking (N = 3644), and the weighted Swiss Mobility and Transport Microcensus 2015 (MTMC, N = 21,399) samples. All samples restricted to 18 to 65 year olds, with the MTMC sample additionally restricted to the Federal Statistical Office’s Commune Numbers present in the MOBIS introduction survey sample

### Response rates

Invitations to the study were sent by post to 90,090 persons. From this sample, 23.70% completed the initial survey. This response rate was likely elevated by the prospect of the 100 CHF incentive for the tracking experiment, mentioned in the invitation letter (even though no incentive was provided for participation in the introductory survey on its own). Only 31.89% of those who completed the introduction survey met the criteria for the field experiment. This was predominately due to the minimal car-use requirement. Many people (age 16 and over) in Switzerland neither have access to a car (22%), nor a drivers license (18%) (BFS and ARE 2017).

The two reminder letters were also effective in the first wave. Of the 5320 who registered, 2397 (45%) did so before a reminder letter was sent, and 1793 (34%) and 1245 (23%) did so after the first and second reminder respectively.

Of those who qualified, 78.06% agreed to participate. This compares similarly to the other studies in Table 3. At the next stage, out of the remaining 5364 participants, 1146 (21.4%) did not start tracking. They either never installed the app, removed it before data was recorded, or were unable to get it to work successfully. Of those who did track, the share with an iOS device was 61%, much higher than the reported 44.4% national market share in 2019 (Comparis 2019), indicating that relatively more Android users were unable or unwilling to use app. Anecdotal evidence from the staff on the study help desk also indicated that more participants had issues installing the app for Android than iOS, and required assistance from the help desk in doing so (Tchervenkov et al. 2020).

Finally, 3690 participants successfully completed the 8-week tracking period, giving a completion rate of 69.4% for those that registered, and 4.06% overall. This is somewhere in the middle of the results from previous studies, with the high incentive appropriately offsetting the long tracking period.

**Table 3:** Response rates in various tracking studies

Project name	MOBIS	SPOT	IN-THE-MOMENT	ATLAS	AKTA	Cincinnati	Atlanta	Reno	Tel Aviv HTS
		(1)	(2)	(3)	(4)	(5)	(6)	(7)	(8)
Tracker	MotionTag	MEILI	rMove	SITSS	Device	Device	Device	Device/App	App
Country	Switzerland	Sweden	USA	NZ	Denmark	USA	USA	USA	Israel
Year	2019	2015	2015	2014	2001–2003	2010	2011	2015–2016	2016–2017
Tracking days	56	7	7	3	112	3	7	7	2
Min. incentive (USD)	$100		$25		Variable	$25	$25	$25	
Validation/annotation	Optional	Yes	Yes	Yes	No	Yes	Yes	Yes	Yes
Invited persons (N)	90,909	130,000	1427	186	25,000	11,118	16,374	25,817	67,199
Intro survey (N)	21,571								
% of invited	23.73%								
Qualified (N)	6,895	1159	478						38,500
% of invited	7.58%	0.89%	33.50%						57.29%
% of intro completed	31.96%								
Registered (N)	5,375	495	295	77	500	4656	1938	602	27,415
% of invited	5.91%	0.38%	20.67%	41.40%			11.84%		40.80%
% of qualified	77.96%	42.71%	61.72%						71.21%
Started tracking (N)	4,218	293	295	73	500	4,656		602	25,201
% of invited	4.64%	0.23%	20.67%	39.25%	2.00%	41.88%			37.50%
% of qualified	61.17%	25.28%	61.72%						65.46%
Completed tracking (N)	3,690	51	240	65	500	3,849	1,061	312	23,240
% of invited	4.06%	0.04%	16.82%	34.95%	2.00%	34.62%	6.48%	1.21%	34.58%
% of qualified	53.52%	4.40%	50.21%						60.36%
% of registered	68.65%	10.30%	81.36%	84.42%	100.00%	82.67%	54.75%	51.83%	84.77%

Taken from (Molloy 2021)

1. Allström et al. (2017), 2. Greene et al. (2012), 3. Safi et al. (2015), 4. Nielsen (2004), 5. Wargelin et al. (2012), 6. Livingston (2011), 7. Stopher et al. (2018), 8. Nahmias-Biran et al. (2018)

### Participant retention

**Fig. 4:**
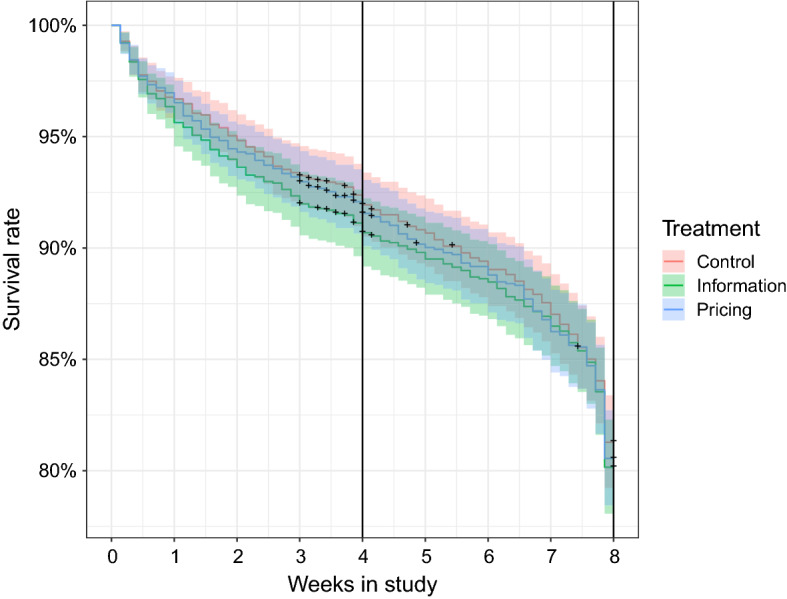
Kaplan-Meier survival curve by treatment group. The cross indicates censoring of participants

To explore the retention rate of participants in the tracking phase, we performed a survival analysis on the duration of tracking in the study. First, a Kaplan-Meier approach (see Fig. 4) shows the impact of the treatment on the length of time which participants tracked. Participants who were automatically dropped out after phase 1 due to poor tracking compliance but were still tracking at the end of phase 1 were censored (marked by a cross). There is no significant difference between the three treatment groups in their survival curves. A sharp decrease in survival is evident in the last study week. As participants were informed at the end of the study that they could delete the app, the last few days of tracking were sometimes not collected before the app was deleted.

Although the participants in the study had a clear participation goal of 8 weeks, after which they would receive the incentive, the survival curve is extremely linear. One would intuitively expect that the attrition rate would be highest early on in the study, and flatten out as participants neared the 8-week goal. This appears to only slightly be the case, with the dropout rate remaining constant throughout the study, even in the second phase. Furthermore, Fig. 4 shows that the treatment did not affect the attrition rate in the second phase.

A time-variant Cox proportional hazards model is estimated to investigate the impact of different factors on the participation duration (see Table 4 for the model results). To account for time-dependent effects, the study period was stratified into fortnightly windows. Those in high-income brackets (more than 12,000 CHF/year) were more likely to stop tracking. Conversely, those from larger households and those with tertiary education were more likely to track for longer. A significant gender-based difference was only observed in the final fortnight, where females were more likely to remain in the study.

**Table 4:** Cox porportional-hazard model

	Beta (SE)	HR (95% CI)	*p*
Income > 12,000 CHF	0.28 (0.09)	1.32 (1.10, 1.58)	0.003**
Household size	− 0.07 (0.03)	0.93 (0.87, 1.00)	0.038*
Age (decades)	0.00 (0.03)	1.00 (0.95, 1.06)	0.883
Tertiary education	− 0.19 (0.08)	0.83 (0.70, 0.97)	0.022*
German speaking	0.03 (0.09)	1.03 (0.87, 1.22)	0.752
Female			
Fortnight = 1	0.02 (0.15)	1.02 (0.77, 1.35)	0.895
Fortnight = 2	− 0.07 (0.20)	0.93 (0.62, 1.39)	0.721
Fortnight = 3	− 0.04 (0.22)	0.96 (0.62, 1.48)	0.841
Fortnight = 4	− 0.28 (0.12)	0.76 (0.60, 0.96)	0.022*
Android			
Fortnight = 1	0.87 (0.16)	2.38 (1.73, 3.26)	0.000***
Fortnight = 2	0.46 (0.22)	1.58 (1.02, 2.45)	0.040*
Fortnight = 3	− 0.01 (0.25)	0.99 (0.60, 1.62)	0.960
Fortnight = 4	0.41 (0.13)	1.51 (1.17, 1.94)	0.002**
Huawei			
Fortnight = 1	0.38 (0.20)	1.47 (0.99, 2.18)	0.057 .
Fortnight = 2	0.37 (0.32)	1.45 (0.78, 2.70)	0.239
Fortnight = 3	0.29 (0.41)	1.33 (0.59, 2.98)	0.487
Fortnight = 4	0.15 (0.21)	1.16 (0.77, 1.75)	0.465
Employed			
Fortnight = 1	− 0.33 (0.16)	0.72 (0.53, 0.97)	0.033*
Fortnight = 2	− 0.07 (0.23)	0.94 (0.60, 1.47)	0.775
Fortnight = 3	0.24 (0.27)	1.27 (0.75, 2.15)	0.369
Fortnight = 4	0.05 (0.14)	1.05 (0.80, 1.38)	0.718
AIC	10484.33		
Concordance	0.602		
Num. events	655		
PH test	0.76		

$$^{***}p<0.001$$; $$^{**}p<0.01$$; $$^{*}p<0.05$$

Contrary to expectations, there was no significant effect of age on the hazard rate. This suggests that common concern about the feasibility of tracking studies for older age groups is unfounded, at least up to the age of 65, the age limit in this study.

The coefficient on employment is also time-dependent. Those in the workforce (i.e. excluding students, homeworkers and retirees) were more likely to remain in the study throughout the first fortnight.

The participant’s mobile device played a much larger role. Having an Android phone of any model increased the hazard drastically. However, this effect was strongest in the first week. The effects were even larger for Huawei models. The incompatibility of GPS loggers with Android (and particularly Huawei devices) is already well known (Montini et al. 2015); however, here the effect is quantified, and seen to be dramatic. The effect was also time-dependent, with the most significant hazard in the first fortnight. At the end of the second fortnight, participants who tracked insufficiently were removed from the study—this explains the reduction in the Android hazard coefficient for the third fortnight, when many of them could have been expected to stop tracking, had they not been removed from the study.

### Post-study retention

**Fig. 5:**
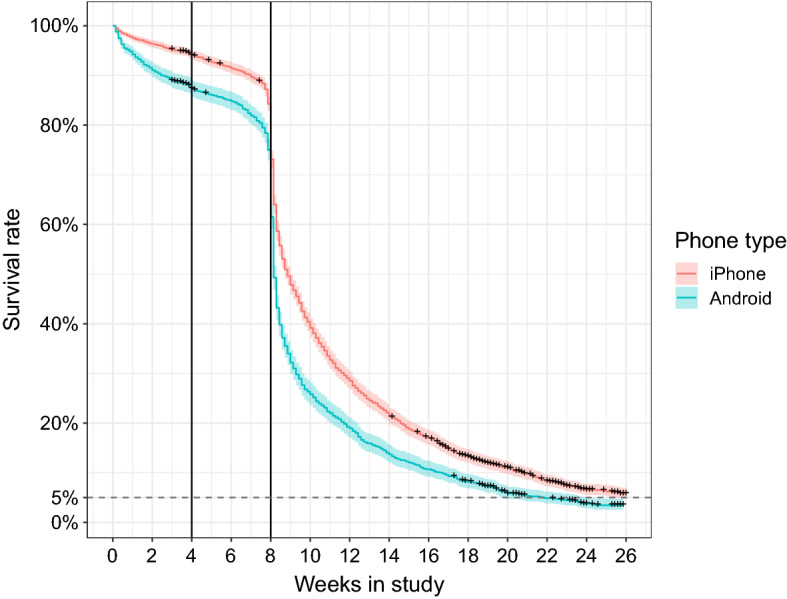
Post-study participation survival curve

At the end of the tracking study, participants were told that they could delete the app, but were also encouraged to continue using it if they wished. Figure 5 shows the dropout rate for the whole study, including the post-study period. The majority of the participants dropped out soon after the study, but even 6 months after the study was completed, around 5% of participants continued to use the app. Anecdotal reports from participants indicated that they enjoyed having an overview of their travel, and that it even continued to inform their mobility decisions. The impacts of the mobile operating system continued even after the study, with the post-study retention rate falling faster for Android users.

### Participant engagement

Participants in the information and pricing groups were effectively treated through information provided in a weekly email detailing their externalities and the costs incurred. Interactions with the emails were recorded using standard email tacking techniques. Emails that remained unopened were effectively missed treatments. Table 5 presents an overview of the engagement with the email communications. The open rate did not change drastically over the duration of the study. Participants in the pricing group viewed their emails much more often than the control or information groups. The information group also opened their emails repeatedly in the first two weeks of phase two, before returning to a pattern similar to the control group, whereas the pricing group continued to repeatedly open their emails.

Participants in the treatment groups likely repeatedly reopened the emails to check their externalities and remaining budget. We suggest that this ‘repeat opening’ behaviour is a useful indicator to measure the level of engagement with the treatment.

**Table 5:** Engagement with various emails through the study

Email & Treatment	n	% Opened	Times opened (mean)	Time to open (h) median (IQR)	
Welcome					
	5475	82.36	2.78	8.50	(2.88–20.33)
Report 1					
	4168	84.88	2.13	7.37	(2.53–19.22)
Report 2					
	4132	81.03	1.87	6.66	(2.59–18.37)
Report 3					
	4105	78.59	1.83	6.19	(2.51–17.85)
Report 4					
Control	1247	79.23	1.62	5.40	(2.30–14.65)
Info	1262	83.68	1.99	5.40	(2.40–16.83)
Pricing	1222	82.90	2.64	6.06	(2.35–17.57)
Halfway					
Control	1250	76.80	1.60	5.60	(2.41–15.54)
Info	1263	83.29	1.72	5.50	(2.53–17.35)
Pricing	1222	80.93	2.17	5.51	(2.24–17.15)
Report 5					
Control	1243	76.43	1.55	5.96	(2.42–15.37)
Info	1255	80.80	1.90	6.28	(2.42–17.29)
Pricing	1213	80.54	2.24	6.94	(2.66–19.82)
Report 6					
Control	1238	77.06	1.87	5.78	(2.35–16.89)
Info	1252	78.12	1.87	5.87	(2.57–17.32)
Pricing	1208	79.22	2.09	6.24	(2.41–17.87)
Report 7					
Control	1235	74.98	1.61	5.83	(2.35–15.83)
Info	1248	77.64	1.66	6.08	(2.44–18.16)
Pricing	1205	80.25	2.02	6.07	(2.33–17.49)
Report 8					
Control	1231	79.69	1.50	6.11	(2.55–17.01)
Info	1246	78.33	1.46	6.41	(2.49–18.85)
Pricing	1200	81.50	2.01	6.55	(2.49–18.80)

### Trip mode and purpose validation

Participants were invited to use the validation interface to confirm the detected mode and purpose of their stages and activities. This was optional, but they were encouraged in the weekly email reports to do so. Even in the second phase, participants were trusted to correct the mode detected by the app. As the mode is crucial in determining the external costs deducted from the mobility budget for the pricing group, this consequently gave them the opportunity to ’game’ the experiment, by for example ‘correcting’ car stages to another transport mode. To test for this, a regression analysis using a zero-inflated negative binomial model was performed with the number of corrections for a day as the dependent variable (see Table 6). A zero-inflated model was used to accommodate the large number of participants who did not correct any stages. While a significant increase in the number of corrections was observed in phase 2, no increase in the number of corrected stages specific to the pricing group was observed. Conversely, the parameters are insignificant but negative. In fact, the information group saw a significant reduction in the corrections in phase 2. One hypothesis is that by receiving more information on their externalities in the weekly reports in the second phase, participants felt discouraged from correcting their stages in the app. Also, no indication was given to participants that they would be penalised for any suspicious behaviour. The fact that no significant change in the average correction rate was seen between treatment groups suggests that the trust in the participants was justified.

**Table 6:** Zero inflated negative binomial model of the validation behaviour

	Count model (1) Corrections/day		Zeros model (2) Correction/day > 0	
Constant	0.744	(0.032)$$^{***}$$	1.504	(0.046)$$^{***}$$
Phase 2	0.047	(0.014)$$^{**}$$	0.050	(0.020)$$^{*}$$
Age (decades)	− 0.024	(0.003)$$^{***}$$	− 0.014	(0.005)$$^{**}$$
Male	0.074	(0.012)$$^{***}$$	0.047	(0.017)$$^{**}$$
Treatment				
Control	–		–	
Information	− 0.029	(0.022)	− 0.053	(0.032)
Pricing	− 0.083	(0.069)	− 0.335	(0.103)$$^{**}$$
Education				
Mandatory	–		–	
Trade/traineeship (baseline)	− 0.098	(0.023)$$^{***}$$	− 0.220	(0.033)$$^{***}$$
Higher education	− 0.014	(0.023)	− 0.321	(0.033)$$^{***}$$
Income (CHF per month)				
Less than 4000	–		–	
4000 $$\le$$ 8000	− 0.134	(0.022)$$^{***}$$	− 0.208	(0.032)$$^{***}$$
8000 $$\le$$ 12,000	− 0.203	(0.022)$$^{***}$$	− 0.324	(0.032)$$^{***}$$
12,000 $$\le$$ 16,000	− 0.230	(0.024)$$^{***}$$	− 0.429	(0.035)$$^{***}$$
More than 16,000	− 0.124	(0.025)$$^{***}$$	− 0.360	(0.038)$$^{***}$$
*Interactions*				
Control * male	–		–	
Information * male	− 0.027	(0.028)	0.139	(0.040)$$^{***}$$
Pricing * male	− 0.004	(0.027)	− 0.001	(0.040)
Pricing * mandatory	–		–	
Pricing * trade/traineeship	− 0.113	(0.057)	0.099	(0.081)
Pricing * higher education	− 0.166	(0.057)$$^{**}$$	− 0.023	(0.082)
pricing * less than 4000	–		–	
Pricing * 4000 $$\le$$ 8000	0.174	(0.059)$$^{**}$$	0.278	(0.084)$$^{***}$$
Pricing * 8000 $$\le$$ 12,000	0.285	(0.058)$$^{***}$$	0.354	(0.083)$$^{***}$$
Pricing * 12,000 $$\le$$ 16,000	0.187	(0.065)$$^{**}$$	0.456	(0.092)$$^{***}$$
Pricing * more than 16,000	0.128	(0.068)	0.368	(0.099)$$^{***}$$
Observations	147,450			
Log Likelihood	− 127,206.400			

$$^{***}p<0.001$$; $$^{**}p<0.01$$; $$^{*}p<0.05$$

In recent years, state-of-the-art machine learning algorithms for mode and activity detection have achieved accuracy rates of over 90%, depending on the approach (Wu et al. 2016; Nikolic and Bierlaire 2017). Hence, we made validation of the activity purpose and stage mode optional for participants, in order to ensure a minimal response burden over the 8 weeks. However, despite this, 85.7% of participants confirmed at least 1 of their trips using the validation functionality; Of this group, 20.4% of iPhone users and 44.1% of Android users did not make a single correction over the 8 weeks. Even with state-of-the-art accuracy rates, it is extremely unlikely that such a percentage had perfect mode and purpose detection. As such, we can assume that these participants did not use or understand the validation interface, and these participants are therefore removed from the following analysis on the mode detection performance. It also indicates that the iPhone validation interface was much more intuitive. To assess the performance of the mode detection, the detected mode was taken as correct if the trip was confirmed but not corrected.

### Mode detection performance

The mode detection provided by the tracking app was a key component of the MOBIS study. As far as the authors are aware, this is the first study to incentivise changes in mobility behaviour based on the output of a mode detection algorithm. As seen in Table 7, the algorithm worked exceptionally well on location data from both operating systems. There is a small difference in accuracy between iOS and Android, with iOS being on average slightly better (92.23% vs. 92.10%) with a *p*-value of 0.01 (test of equal proportions). However, the differences in accuracy are more observable at the categorical level. The iOS performs better on car, local rail, regional rail, tram and walk. However, the differences are only 1–3% in accuracy. Note that ‘Rail’ groups all rail modes together for conciseness. It is also worth noting that while the accuracy of some individual rail modes is quite low, the overall rail accuracy is very good. The main confusion was between different rail mode types.

**Table 7:** Comparison of the MotionTag mode detection performance between iOS and Android

Mode	% Correct			
	Android		iOS	
Airplane	99.48%		98.86%	
Bicycle	81.59%		79.14%	
Bus	66.98%		66.82%	
Car	92.98%		93.15%	
Rail	89.50%		91.05%	
Local train		88.67%		90.18%
Regional train		71.35%		73.40%
Subway		93.56%		92.53%
Train		63.13%		63.78%
Tram	95.01%		96.64%	
Walk	95.56%		97.21%	

Table 8 presents the confusion matrix between the modes for the mode detection algorithm. Here we can see that the algorithm often misdetected car travel as bus travel. For conciseness, the category ‘Other *’ includes those modes which could be manually selected by the participant, but which were not automatically detected. These included: Carsharing, Taxi/Uber, Motorbike/Mopeds, and Gondolas. Most of these were detected as car travel, and the 1500 ‘Bicycle’ trips which were corrected to ‘Other’ were predominately trips by motorbike or moped.

These mode detection results confirmed the indications of our pretest that the automatic detection could indeed be used to calculate the external costs of travel with sufficient accuracy and determine the phase 2 budget and deductions based on these. If the accuracy had been too low, more participants would have dropped out of the study, seeing it as ‘unfair’ if the budget and deductions did not match their travel behaviour.

**Table 8:** Confusion matrix of mode detection accuracy

Predicted	Confirmed mode									
	Airplane	Bicycle	Boat	Bus	Car	Rail	Tram	Walk	Other	Total
Airplane	**2113**	–	–	–	22	–	–	–	–	**2135**
Bicycle	4	**26,201**	136	438	1499	177	149	2771	1500	**32,875**
Bus	1	435	2	**35,713**	15,085	140	280	889	865	**53,410**
Car	372	2495	741	8028	**366,649**	3314	1950	2834	7433	**393,816**
Rail	64	56	85	1748	7298	**60,270**	691	258	298	**70,768**
Tram	–	49	2	128	396	60	**20,174**	149	16	**20,974**
Walk	80	3807	456	1224	9960	868	868	**514,944**	638	**532,845**
	**2634**	**33,043**	**1422**	**47,279**	**400,909**	**64,829**	**24,112**	**521,845**	**10,750**	**1,106,823**

The bold text indicates the correct detection and the row and column totals

### Identified mode detection issues

As previously mentioned, the quality of the mode detection was key to the transport pricing field experiment. A few issues were identified which are worth considering in future studies that apply algorithmic mode detection.

The first consideration concerns those leisure activities that are movement based over a larger area, such as a bike tour, hiking and skiing. Skiing is especially important in alpine areas: In Switzerland, the percentage of the population that ski regularly is 37% (Statistica 2018). Gondolas and chairlifts move at between 15 and 50 km/h, meaning that these trips are often confused with car travel unless the algorithm has been specifically calibrated. On the downhill, skiers reach similar speeds. Taking a strict definition of a transport trip, such movement-based activities should be excluded from the calculation of external costs. If they were to be included, a person could end up being charged for a long hike in the wilderness on the weekend - which would arguably not be in the spirit of a transport pricing scheme.

The second consideration is trip chaining. Shen and Stopher (2014) note that all methods to date (albeit in 2014) did not consider trip chains when detecting the transport mode, and only considered each individual stage. While the mode detection provided by the app was sufficient for the purpose of the transport pricing field experiment, anecdotal evidence indicates that considering trip chains could further improve the performance of the algorithm.

## Conclusion

This work makes multiple contributions to the literature on conducting tracking-based mobility studies, and demonstrates the feasibility of running an incentive-based field experiment using a tracking app. We analysed the effect of the mobile device operating system on GPS tracking studies, and identified certain areas where the difference in OS needs to be considered when undertaking such studies. The impact on participant retention is significant. While this effect is strongest at the start of the study, it persists throughout. The on-boarding of Android users into the study took substantial resources, and we suggest this be accounted for when planning and budgeting such studies. Correspondence by email was effective, and participant engagement did not decline over the 8 weeks. The mode detection algorithm was also sufficiently accurate to support the calculation of external costs in the field experiment. Finally, concerns that participants would manipulate the study by ‘correcting’ their trips in the app were unfounded, with participants adhering to the spirit of the study. Socio-demographic differences in the correction rate do, however, indicate that some participants were more engaged than others.

## Supplementary Information

Below is the link to the electronic supplementary material.

Supplementary file 1 (pdf 112 KB)

## Data Availability

The data is being made available through the ETH Zurich library data archive. Due to the sensitive nature of the tracking data, please contact the project team to discuss access. The archive will also include the tracking data from the post study period, until February 29th, 2020. Data is available on request at the trip level, with personally identifying information is removed, and start and end points aggregated to a 1km grid. To work with higher resolution data (routes and raw GPS tracks) please contact the researchers directly.

## Code availability

The code is available through the following repositories: https://github.com/MOBIS-studyhttps://gitlab.ethz.ch/ivt-vpl/ivt-tracking-web-app/-/tree/motiontag
