# Supplementary material for: The MOBIS dataset: a large GPS dataset of mobility behaviour in Switzerland
Source: Transportation (Amst). 2022 Jun 21:1–25. Online ahead of print. doi: 10.1007/s11116-022-10299-4 (PMC9212210; doi:10.1007/s11116-022-10299-4)
Supplement: Supplementary file 1 — (pdf 112 KB) [file 11116_2022_10299_MOESM1_ESM.pdf]

Appendix

A Introduction survey

1. What is your highest completed level of education?
   - Mandatory education
   - Secondary education (e.g., apprenticeship or diploma)
   - Higher education (e.g., university)
2. What is your age?

   ____
3. What was your citizenship at birth?
   - Swiss
   - Other
   - More than one citizenship (including Swiss)
   - More than one citizenship (not including Swiss)
4. Which country are you a citizen of?

   ____
5. Which is the first country of your dual (or multiple) citizenship?

   ____
6. Which is the second country of your dual (or multiple) citizenship?

   ____
7. What is your current employment status?
   - Employed
   - Self-employed
   - Unemployed
   - Apprentice
   - Student
   - Retired
   - Other
8. Which type of employment do you have?
   - One full-time job (100%)
   - One part-time job
   - More than one part-time job
9. What is your workload? (Percent of a full-time employment)
   - 5%
   - 10%
   - 15%
   - 20%
   - 25%
   - 30%
   - 35%
   - 40%
   - 45%
   - 50%
   - 55%
   - 60%
   - 65%
   - 70%
   - 75%
   - 80%
   - 85%
   - 90%
   - 95%
10. What is the workload of your jobs? (Percent of a full-time employment)
    - Main Job :
    - Secondary job(s) :
    - Total :
11. What is the postcode of your place of employment?

    ____
12. What are the postcodes of your places of employment?
    - Main job : ______________________
    - Secondary job : ______________________
13. Do you own any of the following vehicles?

	Yes	No, but I can arrange to borrow one from someone (e.g., partner, friend, neighbor)	No
Car			
Motorbike			
Bicycle (electric or not)			

14. What kind of fuel does your main car use (the car that use the most) ?

 - Gasoline
 - Diesel
 - Hybrid (gasoline/diesel + electric)
 - Electric
 - Other

15. What is the year of production of your main car?

 - 2015 or later
 - 2011 - 2014
 - 2006 - 2010
 - 2001 - 2005
 - 1997 – 2000
 - 1993 - 1996
 - 1992 or earlier
 - I don't know

16. Which size category applies best to your main car?
 - Small car (e.g. Fiat 500 or Volkswagen Polo)
 - Medium to large car (e.g. Skoda Octavia or Audi A4)
 - Off-road vehicle (e.g. Landrover Discovery)
 - Minivan or van (e.g. Opel Zafira)
 - Luxury car or sports coupé (e.g. Mercedes-Benz E-Class, BMW 7 Series or Porsche 911)
17. What is the engine size of your main car?
 - Less than 1.4L
 - 1.4L - 2L
 - More than 2L
 - I don't know
18. What type of bicycle do you have?
 - Regular bicycle (non-electric)
 - E-bike/Pedelec up to 25 km/h (no license plate)
 - E-bike/S-Pedelec up to 45 km/h (yellow license plate)
19. Do you have a public transport pass? Select all that apply.
 - GA Travelcard
 - Half Fare Travelcard
 - Regional or point-to-point travel card
 - Track 7
 - Other pass
 - No pass
20. How often do you typically use the car as driver, counting weekdays only?

	3 or more days per week	2 days per week	1 days per week	1-3 days per month	Less than 1 day per month	Never
Own Car						
Car sharing service (e.g. Mobility)						

21. How often do you typically use the car as passenger, counting weekdays only?

	3 or more days per week	2 days per week	1 days per week	1-3 days per month	Less than 1 day per month	Never
Car in your household (e.g. with your partner)						
Car-pooling (e.g. with a work colleague)						
Taxi						
App-based service (e.g. Uber, Lyft)						

22. How often do you typically use public transport, counting weekdays only?

	3 or more days per week	2 days per week	1 days per week	1-3 days per month	Less than 1 day per month	Never
Train						
Local public transport (tram, bus, etc.)						

23. How often do you typically use the bicycle, counting weekdays only?

	3 or more days per week	2 days per week	1 days per week	1-3 days per month	Less than 1 day per month	Never
Own non-electric bicycle						
Own electric bicycle						
Bike-sharing						

24. Please indicate for each problem whether it should receive more or less attention from policy makers, compared to how much attention it currently receives.

	Much less attention	Less attention	Neither more nor less attention	More attention	Much more attention	I don't know
Road congestion						
Greenhouse gas emissions from motorized traffic						
Health effects of air pollution from motorized traffic						
Extent of mobility overall (people travel too much)						
Noise from motorized traffic						
Noise from public transport						
Crowding in public transport						
Speeding						
Driving under the influence of alcohol or drugs						
Distracted driving (phone use while driving)						
Accident risk for pedestrians						
Accident risk for cyclists						
Accident risk for drivers						

On the left is a list of factors influenced by transport policy.

25. Please indicate for each factor whether you find its current level to be too low or too high.

	Much too low	Too low	Neither too low nor too high	Too high	Much too high	I don't know
Price of mobility in general						
Capacity of road infrastructure						
Capacity of public transport						
Price of public transport tickets						

26. Please indicate whether you agree or disagree with each policy.

	Strongly disagree	Disagree	Neither disagree nor agree	Agree	Strongly agree	I don't know
Time- and route-specific mobility pricing, made revenue-neutral by lowering other taxes						
Reduction of speed limit from 50 to 30km/h on selected streets						
Dynamic adjustment of speed limits on highways to optimize traffic flow						
Widen major highways with extra lanes						
Creation of more bus-only lanes in cities						
Expansion of cycling infrastructure						
Expansion of car-free zones in cities						
Increasing of the cost of public parking in city centers						
Subsidization of the purchase of electric vehicles						
Permission to pass on the right on highways						
Stricter noise regulations for motorcycles						
Reduction of minimum driving age to 16						
Reduction of the number of public parking spaces in cities						

27. Please indicate your level of agreement or disagreement with the following statements.

	Strongly disagree	Disagree	Neither disagree nor agree	Agree	Strongly agree	I don't know
The government should build sufficient road capacity to satisfy demand at all times						
The government should build sufficient public transport capacity to satisfy demand at all times						
The price for mobility should reflect the social cost (e.g., health, environment, congestion)						
The transport network should be used more efficiently by introducing dynamic pricing (e.g., higher prices during rush hour)						
A journey from A to B should						
always cost the same, regardless of when and which route one travels						
A journey from A to B should cost the same for everyone, regardless of how much they earn						
Public transportation should no longer receive public funds, such that users (rather than taxpayers) cover the full cost						
The government should not intervene in mobility other than providing infrastructure and setting and enforcing traffic laws						
The tax deduction of commuting costs should be stopped (and other taxes lowered to keep overall tax revenue the same)						
The current level of road capacity should not be increased, because more roads lead to more traffic						

28. What is your home postcode in Switzerland?
    - Field/postcode home: ____________
    - Other (I recently moved): ____________
    - I do not live in Switzerland anymore: ____________

29. How many people (including yourself) usually live in your household?
    - 1
    - 2
    - 3
    - 4
    - 5 or more

30. What is your approximate total household income per month? Annual income divided by 12.
    - 4000 CHF or less
    - 4001 - 8000 CHF
    - 8001 - 12000 CHF
    - 12001 - 16000 CHF
    - More than 16000 CHF
    - Prefer not to say

The MOBIS project consists of two parts. You have finished the first part (i.e. this survey) - thank you! The second part of MOBIS is a smartphone study, which tracks participants' mobility using a smartphone app. For participating in this study you, you will receive CHF 100.
Are you interested in participating in the smartphone study?
 – Yes
 – No

31. For which reason(s) do you not want to take part in the smart phone study?
 – I don't want to be tracked
 – I don't want to receive e-mails
 – I don't use a smart phone
 – I don't want to provide my personal data
 – Other (please specify): ____________________

We follow the strictest guidelines in protecting your personal data and we assure you that these data will only be used for research purposes. We anonymise your personal data such that it will not be available to third parties. In addition, we will keep e-mail correspondence to a minimum.

32. Would you reconsider taking part in the smartphone study?
 – Yes, I'm interested in taking part
 – No, I don't want to take part

Thanks for your interest in the MOBIS smartphone study!
To check your eligibility, we need to ask you just a couple more questions.

33. Do you use a smartphone? (Android or iOS/iPhone only)
 – Yes
 – No
34. Do you drive in a professional capacity on all or most days (e.g. taxi driver, train driver, bus driver, tram driver, delivery driver, etc.)?
 – Yes
 – No
35. Are you capable of walking 200m without assistance?
 – Yes
 – No

Thank you very much for completing the survey!

B Registration survey

MOBIS Registration (English)

Welcome to the registration for the smartphone study of the MOBIS project!

Registration requires the following steps:

1. Learn about what this study involves - Provide your email address
2. Read and approve the consent form
3. Download the app and activate it

The MOBIS Smartphone Study

In this smartphone study we will record your mobility behavior over the course of two months. The study conforms to the strictest privacy and confidentiality requirements. Smartphones allow for much more precise research compared to traditional surveys. In addition to smartphone tracking, you will be required to complete an additional final online survey. We will update you weekly on the progress of the study by e-mail. You will be compensated with CHF 100 for your efforts. Detailed additional information about the study can be found here.

To participate in the study you need to provide your e-mail address and consent to the terms in the consent form.

- Please enter your e-mail address. ____________________
- Please re-enter your e-mail address. ____________________
- I agree to participate in the MOBIS smartphone study and consent to the terms in the consent form.
  - Yes
  - No

C Final survey

MOBIS finale questionnaire (English)

1. Were you away from home for at least two days during the MOBIS study period (e.g., on holidays or a business trip)?
   - Yes, once
   - Yes, more than once
   - No
2. When were you away?

	January	February	March	April	May	June	July	August	September	October	November	December
From												
Until												

3. When were you away? Please add up to four more trips.

	January	February	March	April	May	June	July	August	September	October	November	December
Trip From												
Trip Until												
Trip From												
Trip Until												
Trip From												
Trip Until												
Trip From												
Trip Until												

4. Has your employment status changed during the course of the study?
   - Yes
   - No
5. What is your new employment status?
   - Employed
   - Self-employed
   - Unemployed
   - Apprentice
   - Student
   - Retired
   - Other
6. What is the degree of your new employment?
   - One full-time job (100%)
   - One part-time job
   - More than one part-time job
7. What is your new workload? (Percent of a full-time job)
   - 10%
   - 20%
   - 30%
   - 40%
   - 50%
   - 60%
   - 70%
   - 80%
   - 90%

8. What is the workload of your jobs? (Percent of a full-time job)
   – Main Job :
   – Secondary job(s) :
   – Total :
9. What is the postcode of the location of your new employment?

   ____
10. What are the postcodes of the locations of your new employments?
    – Main job : ____________________
    – Secondary job : ____________________
11. To what extent are you able to organise your work schedule?
    – No flexibility (fixed start and end time)
    – Some flexibility (flexible start and/or end time, but completing a set number of hours per day)
    – Full flexibility (flexible start and end time, completing a set number of hours per week, month or year)
12. In your current job(s), do you work from home, at least in part?

    – Yes
    – No
13. How many days a week do you work from home?

    – 1
    – 2
    – 3
    – 4
    – 5
    – 6
    – 7
14. In your current job(s), would you be able to work from home, at least in part?

    – Yes
    – No
    – Don't know
15. How many days a week would you be able to work from home ?

    – 1
    – 2
    – 3
    – 4
    – 5
    – 6
    – 7
    – I don't know
16. How satisfied are you with the Swiss transport system?

	Very dissatisfied	Somewhat dissatisfied	Neither satisfied nor dissatisfied	Somewhat satisfied	Very satisfied
Road infrastructure					
Public transport					

17. Below is a list of potential problems commonly associated with transport. Please indicate for each problem whether it should receive more or less attention from policy makers, compared to how much attention it currently receives.

	Much less attention	Less attention	Neither more nor less attention	More attention	Much more attention	I don't know
Road congestion						
Greenhouse gas emissions from motorized traffic						
Health effects of air pollution from motorized traffic						
Extent of mobility overall (people travel too much)						

18. Below is a list of factors defined by transport policy. Please indicate for each factor whether you find its current level to be too low or too high.

	Much too low	Too low	Neither too low nor too high	Too high	Much too high	I don't know
Price of mobility in general						
Capacity of road infrastructure						
Capacity of public transport						
Price of public transport tickets						

19. Please indicate whether you agree or disagree with each policy.

	Strongly disagree	Disagree	Neither disagree nor agree	Agree	Strongly agree	I don't know
Time- and route-specific mobility pricing, made revenue-neutral by lowering other taxes						
Dynamic adjustment of speed limits on highways to optimize traffic flow						
Widen major highways with extra lanes						
Increase the cost of public parking in city centers						

20. Please indicate your level of agreement or disagreement with the following statements.

	Strongly disagree	Disagree	Neither disagree nor agree	Agree	Strongly agree	I don't know
The government should build sufficient road capacity to satisfy demand at all times						
The government should build sufficient public transport capacity to satisfy demand at all times						
The price for mobility should reflect the social cost (e.g., health, environment, congestion)						
The transport network should be used more efficiently by introducing dynamic pricing (e.g., higher prices during rush hour)						
All people should pay the same for mobility, regardless of when and where they travel						

21. What is the average private cost of your car travel per kilometre?
____________________ Centimes/Rappen

22. Do you agree with the following statements? Compared to public transport, using a car...

	Very much disagree	Disagree	Neither disagree nor agree	Agree	Very much agree
... saves time					
... saves money					
... is harmful for the environment					
... has negative impacts on public health					
... is pleasant					
... is comfortable					
... is convenient					
... makes me flexible/independent					
... allows me to make the best use of travel time					
... protects me from unfavorable weather conditions					
... enables me to transport luggage					
...is safe with regards to traffic					
...can increase congestion					

23. Please indicate your level of agreement with the following statements. The MOBIS study ...

	Strongly disagree	Disagree	Neither disagree nor agree	Agree	Strongly agree
... has affected my travel behavior during the study period					
... will continue to affect my travel behavior in the future					
... has raised my awareness about the external costs of transport					
... has made me consider alternative travel options					
... has made me re-evaluate my car use					

24. What was your motivation to participate in our smartphone study? Please select all that apply.
    - Financial reward
    - Interest in transport policies
    - Interest in impacts of transport (congestion, air pollution, health...)
    - Desire to learn more about personal travel behaviour
    - Other (please specify): ____________________

25. Do you agree with the following statements? The information provided in the MOBIS e-mails ...

	Strongly disagree	Disagree	Neither disagree nor agree	Agree	Strongly agree
... was clear					
... was interesting					
... made me reflect on the content					
... was difficult to understand					

26. How would you define the external costs of your travel behavior?
    - The costs associated with my travel behavior that I have to pay myself.
    - The costs imposed on society as a consequence of my travel behavior.
    - The total costs associated with my travel behavior (sum of private plus societal costs)
    - I don't know what the external costs of travel are
27. Were you aware that you could earn money by changing your travel behaviour during the second phase of the study?
    - Yes
    - No
28. Were you aware that you could reduce the external costs of your travel by changing your travel behaviour during the second phase of the study?
    - Yes
    - No
29. Due to technical reasons, it was not possible to mark a trip as "car-pooled" with someone, even though this is an effective way of reducing external costs. Did you share rides with others in order to reduce your external costs?

    - Always
    - Often
    - Sometimes
    - Rarely
    - Never

    The following questions are about the use of potential revenue from mobility pricing.
30. If dynamic mobility pricing (i.e., prices depending on mode, route and time) were introduced, what should be done with the revenue?
    - The money should be returned to households
    - The money should be used to fund new transport-related projects
    - About half of the money should be returned and the rest spent on transport-related projects
    - Other(please specify) ____________________
31. If the money were returned to households, which option would you prefer?
    - Returning the same amount to everyone (e.g., by lowering health insurance premia)
    - Lowering existing taxes and fees related to motorized transport (e.g., vehicle tax)
    - Lowering existing taxes that are unrelated to transport
    - Lowering public transport fares
    - Other (please specify) ____________________

32. If the money were used to fund transport projects, how should it be prioritized?
    - Projects related to motorized transport
    - Projects related to public transport
    - Projects related to bicycling
    - Projects related to walking
    - No particular mode should be prioritized
33. To what degree do the following statements apply to your lifestyle?

	Does not apply at all	Does not apply	Applies somewhat	Applies fully
I maintain an upscale standard of living				
I live according to religious principles				
I uphold my family traditions				
I enjoy my life to the fullest degree				
I go out often				
I find my life especially pleasing when there is constantly something going on				

34. How often do you engage in the following leisure activities?

	Never	Seldom	Sometimes	Often
Visiting art exhibitions or galleries				
Reading books				
Reading a national newspaper				

35. When you have a really nice dinner in a restaurant, how much do you spend at most per person?
    ______________________CHF
36. Next to each value is a short explanation. Please rate how important each value is for you as a guiding principle in your life.

	Not important at all	Not very important	Somewhat important	Very important	Extremely important
EQUALITY: equal opportunity for all					
RESPECTING THE EARTH: harmony with other species					
SOCIAL POWER: control over others, dominance					
PLEASURE: joy, gratification of desires					
UNITY WITH NATURE: fitting into nature					
A WORLD AT PEACE: free of war and conflict					
WEALTH: material possessions, money					
AUTHORITY: the right to lead or command					
SOCIAL JUSTICE: correcting injustice, care for the weak					
ENJOYING LIFE: enjoying food, sex, leisure, etc.					
PROTECTING THE ENVIRONMENT: preserving nature					
INFLUENTIAL: having an impact on people and events					
HELPFUL: working for the welfare of others					
PREVENTING POLLUTION: protecting natural resources					
SELF-INDULGENT: doing pleasant things					
AMBITIOUS: hard-working, aspiring					

37. In general, how would you say your health is?

- Very good
- Good
- Fair
- Poor
- Very poor
- Prefer not to say
